# Single breath-hold 3D LGE using stack of spiral trajectory

**DOI:** 10.1186/1532-429X-17-S1-Q45

**Published:** 2015-02-03

**Authors:** Yang Yang, Peter Shaw, Jorge A Gonzalez, Christopher M Kramer, Michael Salerno

**Affiliations:** 1 Biomedical Engineering, University of Virginia, Charlottesville, VA, USA; 2 Radiology, University of Virginia, Charlottesville, VA, USA; 3 Medicine, University of Virginia, Charlottesville, VA, USA

## Background

Late gadolinium enhancement (LGE) imaging is the gold standard for noninvasive evaluation of myocardial scar. In the clinical setting, the standard imaging protocol involves a breath-held segmented inversion-prepared 2D Cartesian acquisition at a single location. This breath-hold scan is repeated 10 -14 times to cover the entire left ventricle. Although multislice 2D LGE has shown great diagnostic accuracy, the multiple breath-holds increase the scan time to approximately 10min and patient fatigue which can result in poor breath-holding and ghosting artifact. In this study, we propose to perform the 3D LGE imaging in a single breath-hold using a stack of spiral trajectory.

## Methods

Single breath-hold 3D stack of spiral LGE images covering the entire LV were acquired in 27 subjects undergoing clinical scans following the conventional multisilce 2D LGE on a Siemens 1.5T Avanto scanner. The 3D spiral LGE sequence consisted of 12 partitions of a dual density spiral trajectory. Each spiral readout was 4ms long with 24 interleaves to support 1.5x Nyquist in the center and 0.7x Nyquist in the edge of kspace. At each cardiac cycle, 2 out of 24 interleaves were acquired for each partition resulting in an acquisition window of approximately 160ms. All of the spirals were acquired in a single 12 heart beat breath-hold. Other sequence parameters included: TR 7ms, TE 1ms, TI 300~400ms, FA 20^o^, FOV 340mm, in-plane resolution 1.5mm, 12 slices, and thickness 8mm. The images were reconstructed using SPIRiT. Image quality was rated on a 5 point scale (1- very poor to 5 excellent) by two cardiologists.

## Results

**Figure 1:**
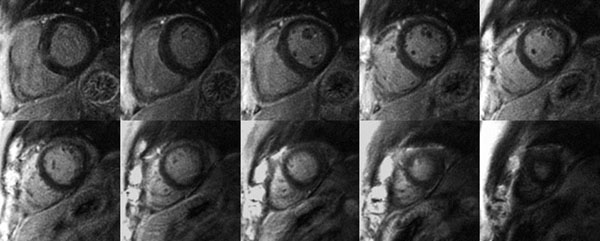
3D spiral LGE images from a subject without scar

**Figure 2:**
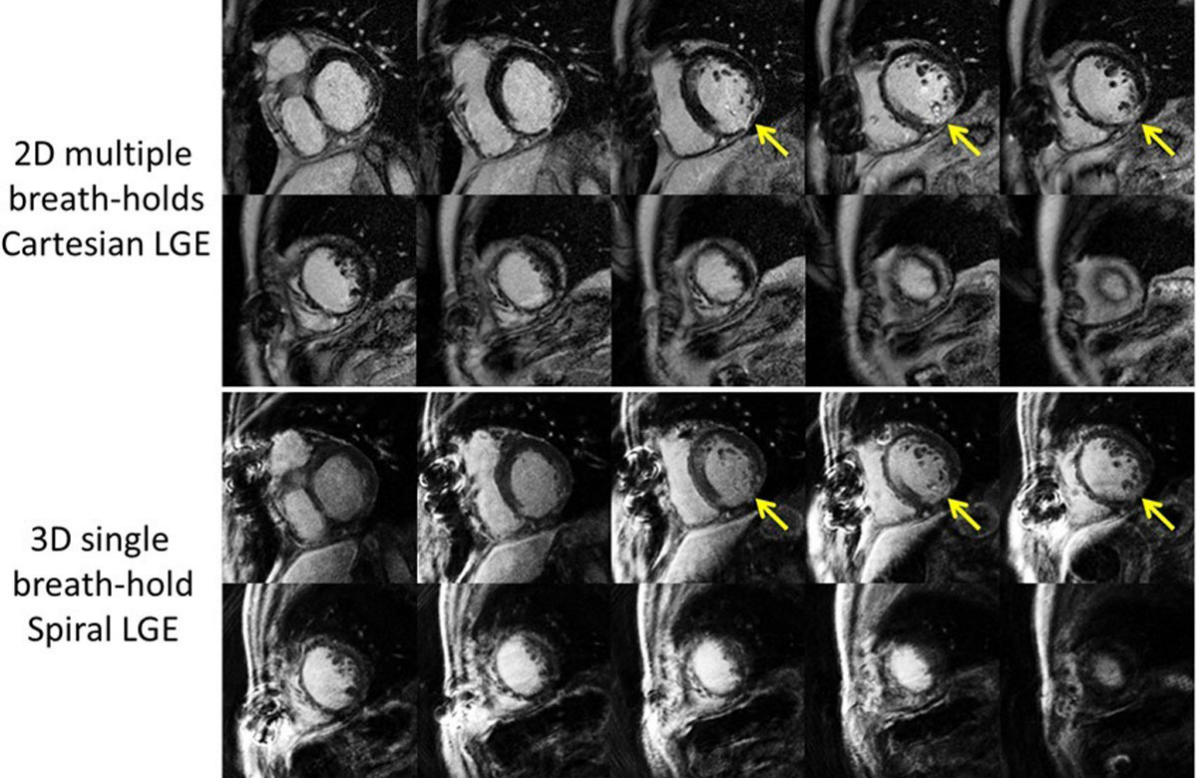
Top two rows: multiple breath-hold 2D LGE images. Bottom two rows: single breath-hold 3D spiral LGE images. Arrows: the myocardial infarction in the inferior wall.

Figure 1 shows the typical negative 3D LGE images from a subject without any scar. Figure 2 shows the multislice 2D LGE images (top two rows) and 3D LGE images (bottom two rows) from patients undergoing a viability study. Both of the 2D and 3D LGE images show a myocardial infarction in the inferior wall. Although the 3D LGE images were acquired within one breath-hold, the SNR of the images were still adequate for diagnostic purpose. The multislice 2D LGE scan time required approximately 10min while the 3D LGE scan time took only 10s. The average image quality score was 3.7 from cardiologist 1 and 3.6 from cardiologist 2.

## Conclusions

We demonstrate the successful application of single breath-hold 3D LGE imaging using stack of spiral trajectories. As with the standard 2D multislice LGE images, 3D LGE images are able to differentiate myocardial scar, while the scan time is dramatically reduced. Such an approach will improve patient throughput in CMR.

## Funding

K23 HL112910-01.

